# Assessment of Vehicle Dynamic Behavior Under Piezoelectric Actuation via Simcenter AMESim Modeling †

**DOI:** 10.3390/mi16101087

**Published:** 2025-09-26

**Authors:** Nezha Chater, Ali Benmoussa, Benaissa El Fahime, Mohammed Radouani

**Affiliations:** Laboratory of Mechanic, Mechatronic and Command, Moulay Ismail University of Meknes, P.O. Box 298, Meknes 50000, Morocco; ali.benmoussa@edu.umi.ac.ma (A.B.); b.elfahime@ensam-umi.ac.ma (B.E.F.); m.radouani@ensam-umi.ac.ma (M.R.)

**Keywords:** piezoelectric actuator, suspension system, energy harvesting, multi-physical modeling, Simcenter AMESim software

## Abstract

Recent research has focused on energy recovery and storage technologies. One of the materials allowing the recovery of dissipated energy is the piezoelectric material (PE). These functional materials perform reversible energy conversion, transforming electrical energy into mechanical and vice versa. In this study, we investigate the recovery of vibratory energy in vehicle suspension systems—energy traditionally dissipated by conventional shock absorbers—using piezoelectric materials to capture this wasted energy and redirect it to the vehicle’s auxiliary power supply network. We propose an integrated electromechanical model incorporating piezoelectric actuators in parallel with the suspension mechanism. The collected energy is processed and stored for later use in powering accessories such as windows and mirrors. The idea is to integrate renewable energy sources to optimize the performance of the vehicle. We proposed a Multiphysics model of the system under a software used to this type of modeling (Simcenter AMESim v1610_student). The simulation results of the system and its various sub-systems are presented for studying the piezo-actuator response to reduce consumption and increase energy performance in a vehicle. These findings will undergo experimental validation in the project’s subsequent phase.

## 1. Introduction

Reducing overall power consumption requires improving fuel economy, reducing emissions and meeting the power demand of additional subsystems in a vehicle [1]. Consequently, recovering the energy dissipated by the shock absorbers is potentially advantageous. Piezoelectric materials have been actively studied in energy recovery applications for the past few decades. This is due to the advantages presented by these types of materials, which is mainly their high actuation frequency range, their relatively high-power density and their bidirectional coupling between mechanical and electrical properties [2,3,4]. 

The piezoelectric effect exists in two areas. On the one hand, the ability of the material to transform mechanical deformation into electrical charge, which represents the direct piezoelectric effect; on the other hand, its ability to convert an applied electrical potential into mechanical strain energy, which is the opposite effect. This property gives these materials the ability to absorb mechanical energy, generally ambient vibrations, and transform it into electrical energy that can be used to power other devices. In other research, piezoelectric devices are embedded in the acoustic chamber [5] on the tire rim [6] or in the central face of the wheel [7]. Authors of other works chose to bond the piezoelectric energy harvesters on the outside surface of the tire as stated in [3,8], the other inside the tire [9,10]. In our case the piezoelectric beam is attached to the un-sprung mass. The model proposed includes the integration of the piezoelectric material in four wheels of the vehicle unlike other works which only concerns the quarter of a car. 

This article is organized as follows: Section 2 presents the operating principles and electromechanical behavior of piezoelectric actuators. Section 3 introduces the multiphysics modeling framework, including both quarter-car and full-vehicle configurations with integrated piezoelectric elements, developed in Simcenter AMESim. Section 4 focuses on practical implementation aspects, covering signal conditioning techniques and energy storage solutions required for stable power delivery. Section 5 outlines the experimental methodology, including instrumentation, excitation protocols, and data analysis procedures for model validation. Finally, Section 6 summarizes the main results and proposes directions for future integration of piezoelectric harvesting systems in real-world automotive applications.

## 2. Model Description: Material and Effects

### 2.1. Piezoelectric Material

Of Greek origin, the term ‘piezoelectric’ means ‘to press,’ and this phenomenon was discovered in 1880 by the French physicists Pierre and Jacques Curie [11]. Piezoelectric materials have a crystalline structure with ionic bonds and exhibit anisotropic properties; they depend on the direction of the applied force and the orientation of the polarization electrodes. Their special feature is that they can work both ways: they can detect pressure and create movement when electricity is applied. In its neutral, unstressed state, the crystal has balanced dipole moments. The application of mechanical stress deforms these dipoles, generating an electric charge (direct effect). Conversely, the application of an external electric field creates a charge imbalance, inducing mechanical stress or deformation (reverse effect). The electromechanical behavior of these materials is described by the following constitutive Equations (1) and (2):

(1) $$D_{i}=d_{ij}\sigma_{j}+\varepsilon_{ij}^{T}e_{i}$$

(2) $$\delta_{j}=s_{ij}^{E}\sigma_{j}+d_{ij}e_{i}$$

where

*i* = 1, …, 6: components of the mechanical strain tensor;
*j* = 1, 2, 3: components of the electric field vector.
*D*: Electric charge density (C/m^2^),
*d*: Piezoelectric charge constant (m/V or C/N),
*σ*: Applied mechanical stress (N/m^2^),
*ε*: Permittivity of the piezoelectric element (F/m),
*e*: Electric field (N/C),
*δ*: Mechanical strain,
*s*: Elastic compliance coefficient of the piezoelectric element (m^2^/N).

**Figure 1:**
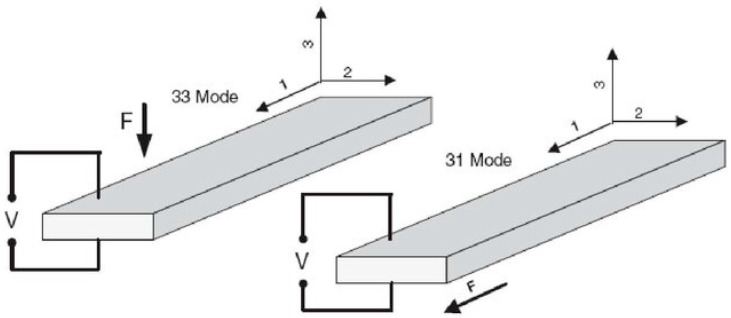
Schematic representation of the piezoelectric modes 33 and 31, illustrating the directions of polarization, mechanical stress, and voltage generation [13].

Two coupling modes dominate the use of piezoelectric materials (Figure 1). Mode 31, which is particularly well suited to energy recovery systems, especially cantilever beams [12], optimizes the coupling of lateral stresses. Conversely, mode 33—which generates higher power levels—is preferred for high-power applications (automotive, industrial machinery) [4]. 

**Figure 2:**
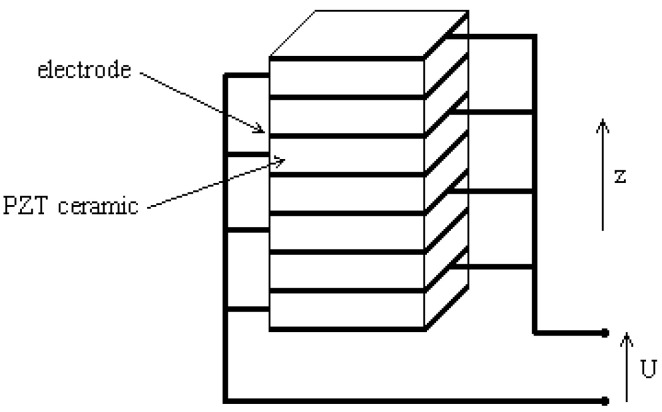
Wafers of piezoelectric material sandwiched between electrodes.

The standard configurations for energy recovery remain the bimorph (mode 31) and the stack (mode 33). A piezoelectric stack is a multilayer piezoelectric foil stacked one on top of the other (Figure 2). These layers (represented by *n_w_*) are connected mechanically in series and electrically in parallel. The piezoelectric beam operates in mode 33 where the mechanical force is applied along the polarization axis while the electrical charge is collected on the surface perpendicular to the polarization axis. Stacks are preferable for use in low frequency ranges and high force excitation [14].

The geometry of the stack helps to amplify charge generation compared with a single layer. Equation (3) shows the relationship between the number of piezoelectric layers *n_w_*, the force *F* and the voltage *V* with the stack output current *Q_stack_* [15]:

*Q*_*stack*_ = *n_w_*
*d*_33_*F* + *C*_*p*_
*V* (3)

where

*Q*_*stack*_: Total charge generated by the piezoelectric stack (Coulombs C);
*n_w_*: Number of wafers;
*d*_33_: Direct piezoelectric coefficient (axis 3 in direction 3) (C/N);
*F*: Longitudinally applied mechanical force (N);
*C*_*p*_: Electrical capacity of the stack (Farads F);
*V*: Applied or generated electrical voltage Volts (V).

**Figure 3:**
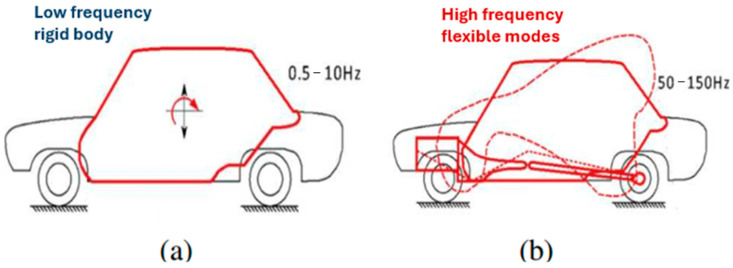
Schematic representation of the main vibratory zones in a motor vehicle [8]: (**a**) Rigid body movements (bounce, pitch, roll), typically located between 0.5 and 10 Hz; (**b**) Flexible modes of structure and wheel-suspension interaction, mainly located in the 50 to 150 Hz band, an area of strategic interest for piezoelectric harvesting.

The use of energy harvesting systems in cars takes advantage of various natural vibrations, from low to high frequencies. In our study, we focus on the frequency range of 0 to 150 Hz, which captures a lot of the vibrational energy in vehicles. This range includes the main vibrations related to the car’s body, suspension, and how the tires interact with the road, making it especially useful for collecting energy and reducing vibrations using piezoelectric technology [10,16] (Figure 3).

### 2.2. Modeling Approach Description

To implement a block diagram model of the vehicle, we use AMESim (Advanced Modelling Environment for performing simulations) from Simcenter International, which is a dynamic simulation environment of various engineering fields: mechanical, electrical, thermal, and physical multi-domain systems [17]. 

**Figure 4:**
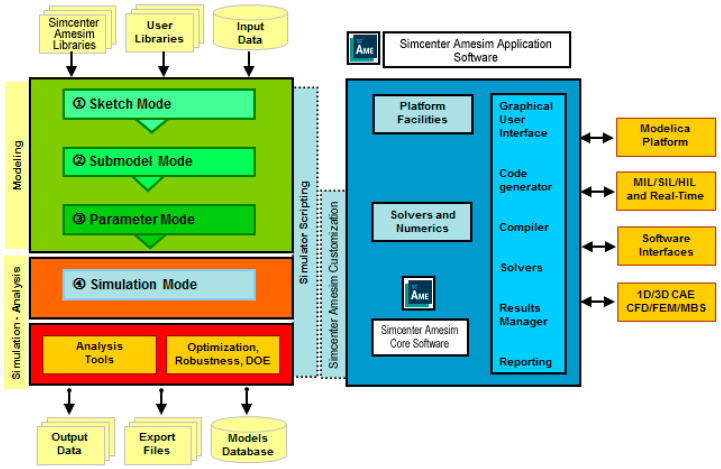
Simcenter Amesim functional architecture (simplified view).

The modeling environment is based on a multi-domain/multi-level approach (Figure 4). It provides a comprehensive system simulation through an intuitive graphical interface, displaying the system throughout the entire simulation process.

### 2.3. Quarter-Car Model with Piezo-Actuator

Piezoelectric actuators are used in a wide range from mechatronic applications like fuel injection to the field of smart structures for micro-positioning or noise and vibration suppression. The mechanical vibrations of the Car suspension system (CSS) are the most widely explored field of study. This system attenuates the disturbances induced by road irregularities to ensure maneuverability, stability and passenger comfort. 

**Figure 5:**
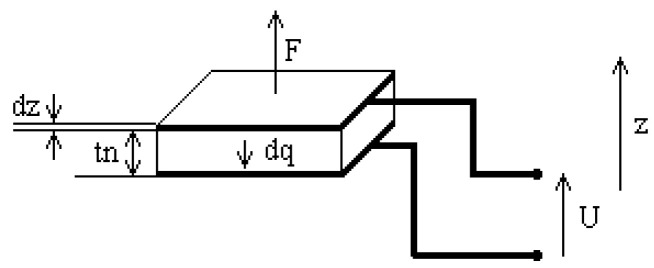
Characteristics of a linear piezoelectric stack actuator.

The piezoelectric actuator used in this study is composed of several ceramic layers, which are electrically connected in parallel and are mechanically connected in series shown in Figure 1. The piezoelectric stacks are made of Lead Zirconate Titanate PZT-5H (Pb[Zr_x_Ti_1−x_]O_3_), a soft piezoceramic known for its high dielectric constant and strong electromechanical coupling, widely employed in energy-harvesting and Microelectromechanical Systems (MEMS) devices [18,19]. It is characterized, as presented in Figure 5, by the number of wafers, the area of a wafer and the thickness of a wafer.

Equation (4) describes the mechanical displacement generated by the piezoelectric actuator as a function of its intrinsic parameters, including the piezoelectric coefficient *d_33_*, the relative dielectric permittivity *εr*, and the geometric dimensions of the stack. This relation forms the foundation of the electromechanical coupling used in the system modeling:

(4) $$dz=\frac{s_{33}^{E}\cdot n_{w}\cdot t_{n}}{A}\cdot F+n_{w}\cdot d_{33}\cdot U$$

where

$s_{33}^{E}={2.18\times 10}^{-11}$ m^2^/N: Elastic constant (reverse of Young modulus);
*n_w_*: Number of wafers;
*t_n_*: Thickness of a wafer;
*A*: Area of a wafer;
*F*: Input force;
*U*: Input voltage;
$d_{33}=k_{33}.\sqrt{\varepsilon_{0}\cdot \varepsilon_{33}^{T}\cdot s_{33}^{E}}$: Piezoelectric constant multiplied by the electromechanical coupling factor $k_{33}=7.72{\times 10}^{-1}$;
$\varepsilon_{0}=k_{33}.\frac{1}{36\pi \times 10^{9}}\;F.d/m$: Vacuum permittivity;
$K_{33}^{T}={2.9\times 10}^{3}$: Relative permittivity.

The piezoelectric actuator parameters applied in our analysis are detailed in Table 1.

Table 2 and Table 3 summarize the main mechanical and electrical data of the piezoelectric systems employed in our model (sourced from Piezo Systems, Inc., Woburn, MA, USA).

The Quarter Car Model (QCM) widely used for its simplicity and its ability to capture the essential characteristics of the system, has received particular attention in the literature. For example, Vaishnav et al. [20] derived a transfer function representation using Cramer’s rule; Sharma et al. [21] modeled a QCM with two degrees of freedom in state space (MATLAB).

**Figure 6:**
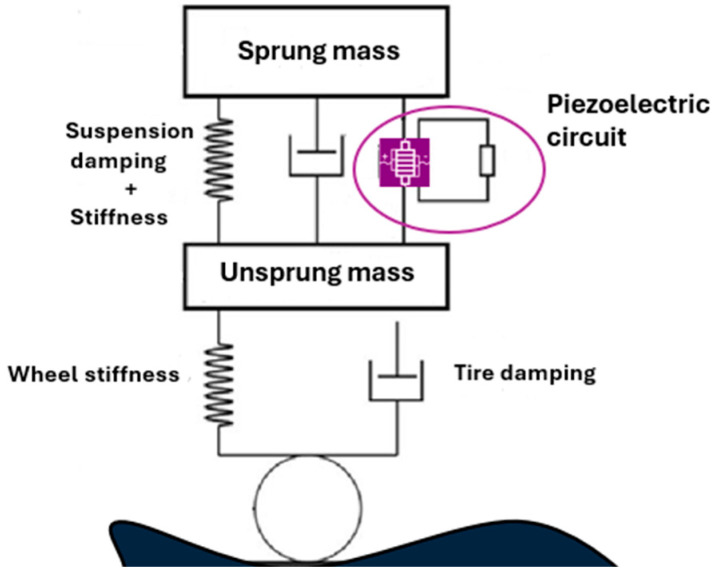
Quarter-car suspension system integrating a piezoelectric actuator.

The QCM is well adapted to experimental modeling, its most common configuration incorporating two moving plates (representing the sprung and unsprung masses) connected by springs and dampers. The variations between models lie mainly in the representation of the unsprung mass (simplified plate or real tire) and the type of excitation applied. The suspension system of the quarter-car (piezoelectric actuator, sprung mass, unsprung mass, suspension and tire) is presented in Figure 6. It shows the way the piezoelectric component is fixed in the wheel.

## 3. Multi-Physical Models and Simulation Result

### 3.1. Multiphysics Quarter-Car Model

**Figure 7:**
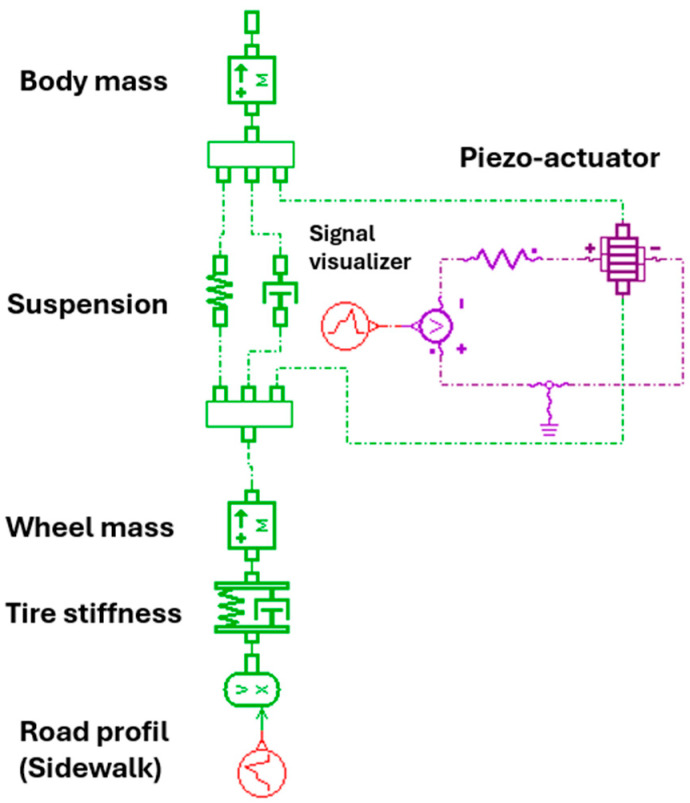
Piezoelectric Quarter-Car Suspension Model in Simcenter AMESim.

To consider a simplified representation of the dynamic behavior of a vertical suspension, a Quarter Car model is proposed in Figure 7.

The piezo actuator is integrated into the suspension system, between the non-suspended mass (wheel) and the suspended mass (chassis). It is mounted in parallel with the traditional suspension element. When road irregularities (road profile) induce vibrations, the mechanical deformations undergone by the piezo actuator generate an electrical signal (piezoelectric effect). This signal, proportional to the forces, is then displayed and analyzed by the Signal visualizer to monitor vibration activity and optimize damping.

A comprehensive overview of the simulation parameters employed in this model are provided in Table 4.

**Figure 8:**
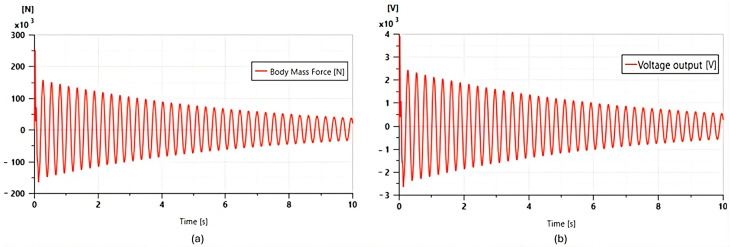
Piezoelectric response measured in the quarter-car model: (**a**) Applied force; (**b**) Generated voltage.

These parameters were chosen based on other works from the literature [22]. The results of the Multiphysics simulation of the vehicle quarter model are presented below, with the force (Figure 8a) and the voltage (Figure 8b) plotted as functions of time.

This figure depicts the behavior of the piezoelectric actuator under the mechanical excitation induced by the displacement of Car body mass.

- Applied force (a): The graph shows the temporal evolution of the mechanical force exerted on the piezoelectric actuator. The waveforms reflect the dynamic disturbances induced by the road profile, with peaks corresponding to shocks and irregularities.
- Simulated voltage (b): the graph shows the electrical output signal (in volts) generated by the piezoelectric actuator in response to the force applied to the mass. The curve shows a clear temporal proportion with the force graph: each force peak immediately produces a voltage peak, demonstrating the direct piezoelectric effect.

These results confirm that the piezoelectric actuator in the suspension works well as a sensor that converts mechanical vibrations (forces) into a useful electrical signal (voltage). The amplitude and shape of the signals give an idea of the quantities for the analysis of suspension stress and recoverable energy.

**Figure 9:**
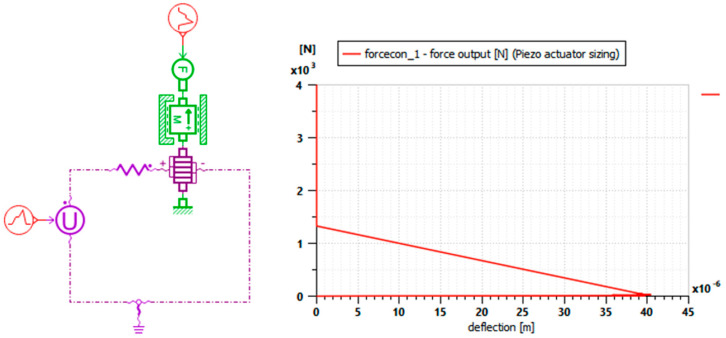
Force input versus deflection of piezo actuator.

Performances of a piezoelectric actuator are partially given by plotting the force input versus deflection (Figure 9).

This curve provides a means to validate the actuator’s design. Specifically, a deflection of 35 µm corresponds to an applied force of approximately 150 N, confirming the adequacy of the selected configuration.

### 3.2. Global Model

**Figure 10:**
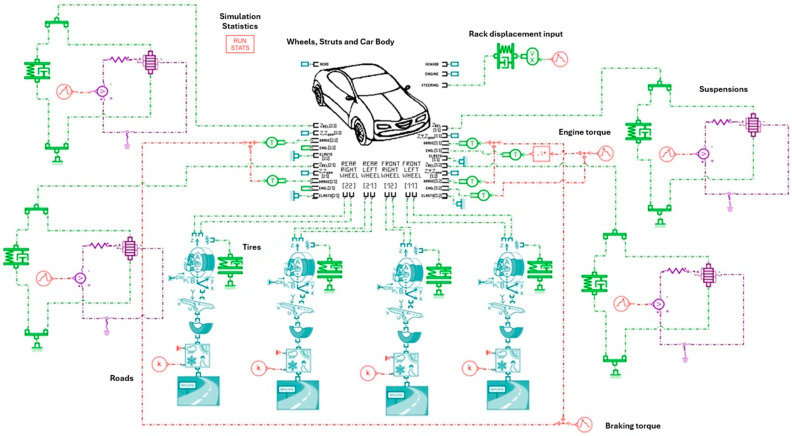
Simcenter AMESim Model of dynamic vehicle with PE [23].

This section focuses on the implementation of the global model. The chassis sub-model serves as the core of the vehicle dynamics architecture, to which all other subsystems can be connected (suspension–spring, damper, stop, anti-roll bar–aerodynamic module, tire, road, sensors, engine, brakes, steering system, powertrain module, external load, etc). The interconnection between these sub-models, including the piezoelectric actuator, is illustrated in Figure 10.

The chassis model used is a 15 DOF model. At the center of the diagram in Figure 10 is a block representing the vehicle body and its connections to the four wheels. This central block integrates the various subsystems: “body”, “steering rack”, “axle”, “wheel” and all the mechanical articulations between these elements. It receives mechanical inputs such as vertical displacement due to suspension, engine torque and steering signals, and transmits forces to the wheels.

**Figure 11:**
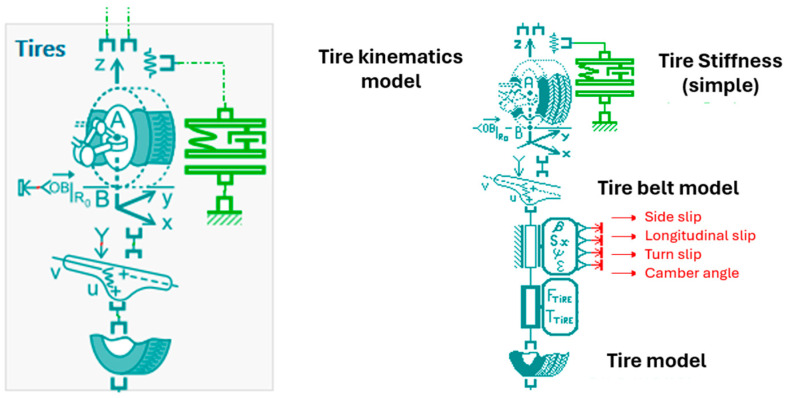
Detailed tire and suspension model illustrating the wheel–road interaction, suspension compliance, steering linkage, and tire deformation for dynamic and vibro-acoustic analysis.

Figure 11 represents a detailed tire model used in vehicle dynamics simulations. It includes a multi-body representation of the wheel–suspension assembly and its interaction with the road surface. The tire is modeled with degrees of freedom in translation (x, y, z) and rotation, allowing accurate representation of wheel dynamics. The vertical compliance is defined by a suspension spring–damper system connected to the chassis. Steering motion is applied via the steering linkage, and the contact forces at the road interface are computed based on tire deformation and road profile input. This model allows the analysis of ride comfort, road holding, and vibro-acoustic behavior.

**Figure 12:**
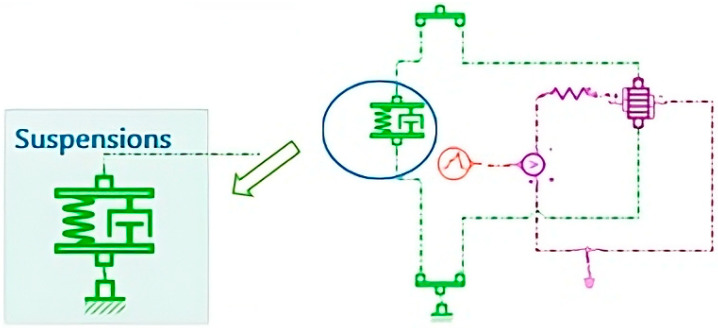
“Suspensions” Block: Suspension model with integrated piezoelectric actuator. This block represents a simplified suspension model combining a spring, a damper, and a piezoelectric actuator. It is used to study the system’s dynamic behavior and the conversion of mechanical vibrations into electrical energy.

As shown in Figure 12, the suspension block serves as a reference for the modeling approach employed in this study, illustrating the key components considered in the dynamic simulations. 

**Figure 13:**
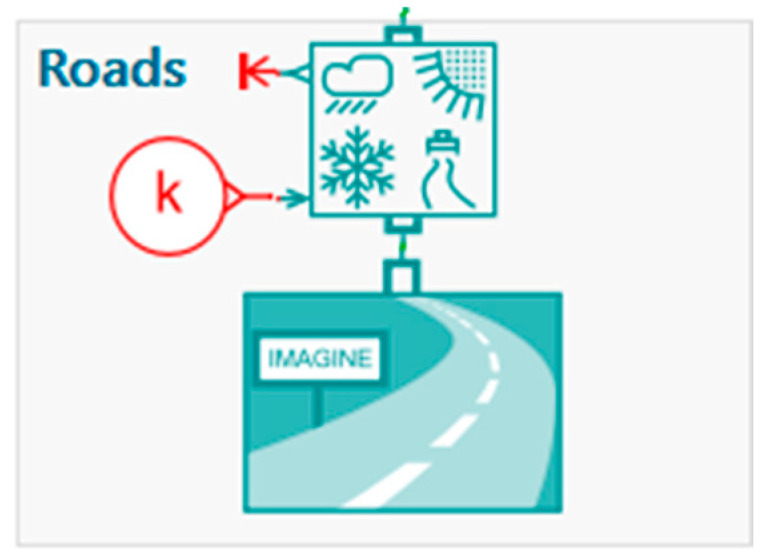
“Roads” Block: Generation of road profiles based on climatic and topographical conditions. This block (simulates road irregularities based on various parameters (rain, snow, roughness, etc.). It provides a realistic vertical excitation profile to the suspension system, used to test dynamic response and energy harvesting capability.

As shown in Figure 13, the input to the suspension system includes realistic road excitations derived from varying environmental and surface conditions. These excitations serve as a basis for evaluating the suspension’s dynamic performance and energy harvesting efficiency.

**Figure 14:**
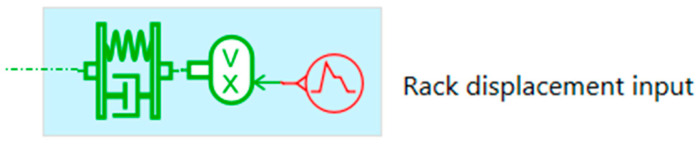
Rack displacement input simulation block, illustrating a mass–spring–damper system, a sensor for displacement and velocity, and a defined excitation source for system dynamics evaluation.

The rack displacement input simulation block represented in Figure 14 applies a predefined displacement profile to the steering rack or a linear actuator model, enabling controlled excitation of the mechanical system. It integrates a mass–spring–damper mechanical subsystem, a sensor providing real-time position *x*(*t*) and velocity ẋ(t) and a signal source defining the excitation profile. The system’s dynamic behavior is modeled using the classical Equation (5):

(5) mẍ(t)+cẋ(t)+kx(t)=F(t)

where *m* is the mass of the moving component, *c* is the damping coefficient, *k* is the stiffness of the spring element, *x*(*t*) is the displacement, ẋ(*t*) is the velocity, ẍ(*t*) is the acceleration and *F*(*t*) is the external force applied by the excitation signal.

This configuration is suitable for vibro-acoustic analysis, structural durability studies, and validation of control strategies.

**Figure 15:**
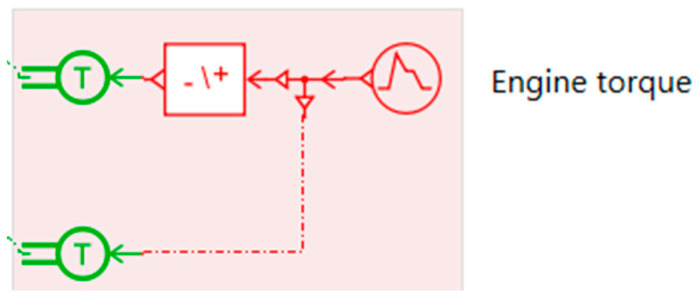
Engine Torque Input. This block represents the engine torque source applied to the powertrain. The signal generator provides a customizable torque profile, which is fed into the system to simulate real driving conditions. The torque is transmitted through the drivetrain to the wheels, enabling the analysis of propulsion dynamics, vehicle acceleration, and its interaction with mechanical or energy recovery components.

The engine torque input used in the simulation is depicted in Figure 15. It enables the analysis of vehicle dynamics under various driving scenarios.

**Figure 16:**
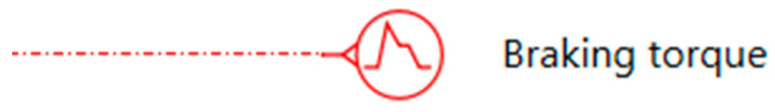
Braking Torque Input. This symbol represents the braking torque source applied to the wheel hub. It allows a time-varying braking profile to be imposed in the model to analyze its effects on vehicle dynamics, load transfer, and interaction with energy harvesting systems.

Figure 16 presents the application of braking torque in the simulation, which allows investigation of the vehicle’s response and load transfer during braking.

**Figure 17:**
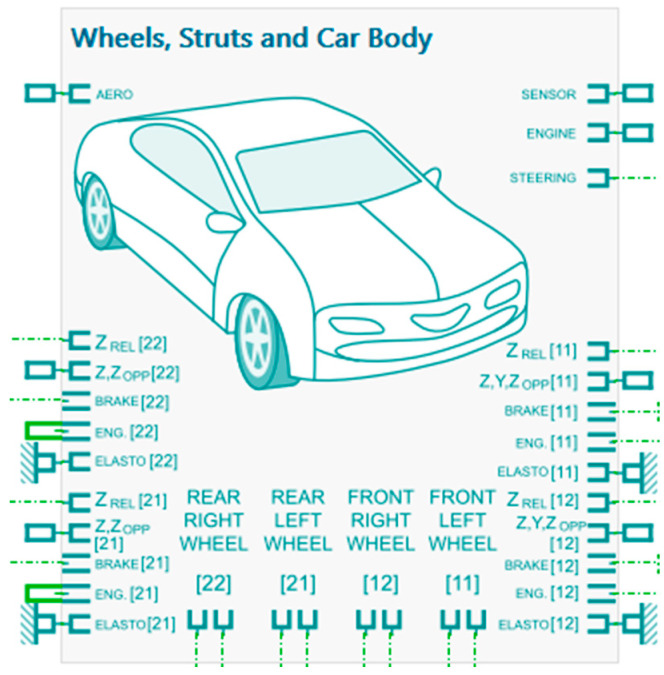
Schematic vehicle model in Simcenter AMESim, linking the car body, suspensions, and four wheels, with the main mechanical interactions and connections to sensors, engine, steering, and aerodynamics modules. where: Z_REL_: Vertical relative displacement (suspension travel) between the wheel and the car body; Z,Z_OPP_: Components of vertical (Z), lateral (Y), and differential (opposite/OPP) displacement; BRAKE: Brake system input signal (braking command); ENG.: Engine input, typically representing torque or force transmitted to the wheel; ELASTO: Elastic element (passive suspension component such as spring or damper); SENSOR: Sensor outputs (e.g., accelerometers, displacement sensors); ENGINE: Global engine module; STEERING: Steering system module; AERO: Aerodynamic forces applied to the vehicle body; [11] = Front left wheel; [12] = Front right wheel; [21] = Rear left wheel; [22] = Rear right wheel.

An overview of the integrated vehicle model and its main subsystems within Simcenter AMESim is provided in Figure 17.

The Global model in Figure 10 is used to analyze the geometric behavior of the vehicle under different inputs without considering complex dynamic effects, making it an effective teaching or pre-study tool. The purple-highlighted components represent the piezoelectric transducer circuits integrated into the system at the location of all four suspension units.

Simulation of the overall model was conducted based on the parameter set presented in Table 5.

The values attributed to these parameters typically correspond to an average compact light vehicle and are chosen to be representative of most sedans or compact internal combustion/hybrid vehicles. They are taken from manufacturer specifications and ISO/SAE standards.

The following figures display the time-domain response of the piezoelectric actuator integrated into the suspension system, illustrating its contribution to the vehicle’s overall dynamic performance.

**Figure 18:**
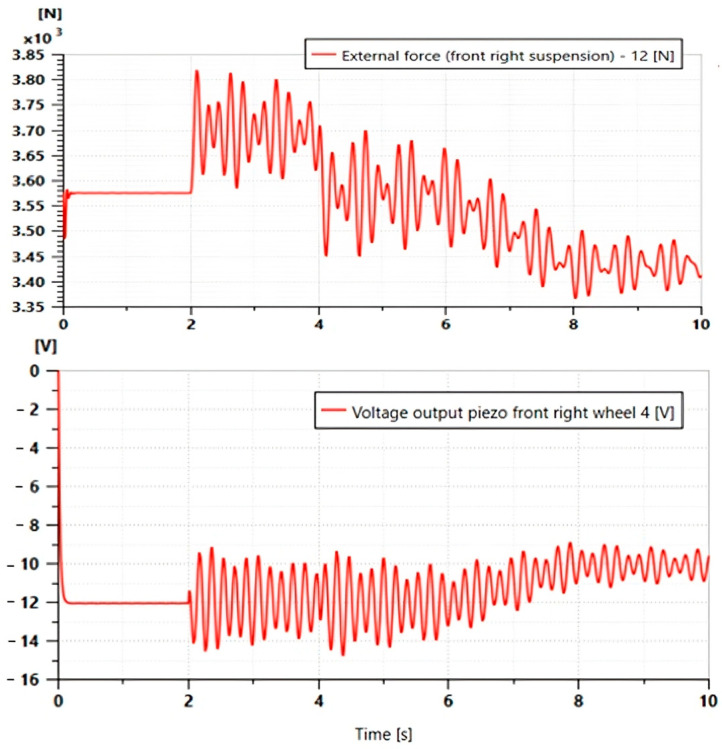
Simulation results: voltage and force outputs at piezo actuator.

The graph (Figure 18) captures the dynamic response of a vehicle’s right front suspension to a sharp vertical excitation simulating a pothole-type disturbance. The external force (upper curve, scale in kN) shows a sharp drop from 3.85 kN to 3.35 kN, followed by damped oscillations around 3.6 kN, revealing energy dissipation by the shock absorber. At the same time, the piezoelectric voltage (lower curve, V scale) generated on the wheel responds with a negative peak at −16 V during rapid decompression, then oscillates in inverse phase with the time derivative of the force. This negative correlation ($V\propto -\frac{dF}{dt}$) perfectly illustrates the piezoelectric law, where the sensor converts rapid variations in mechanical load (suspension unloading) into electrical signals that can be used for diagnostics or energy recovery.

**Figure 19:**
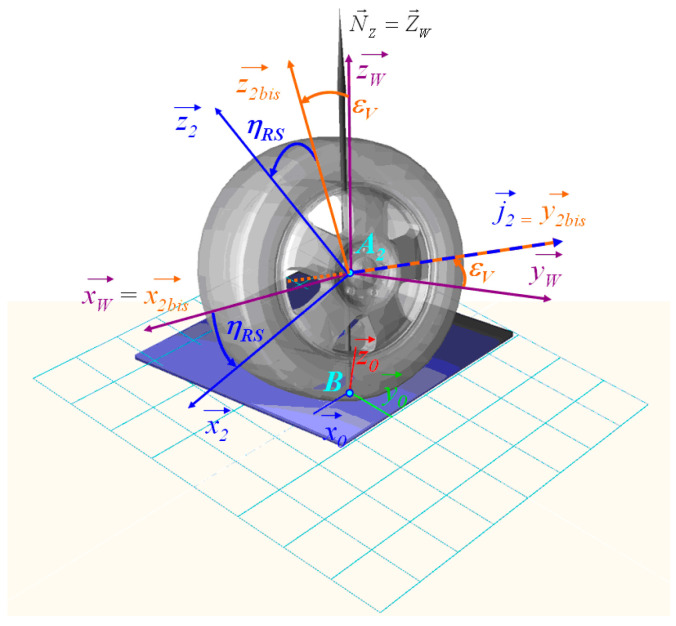
Representation of kinematic reference frames and key points in the car tire model.

To better understand the dynamic behavior of the wheel and suspension system, it is essential to analyze the kinematics at key points such as A_2_. Figure 19 illustrated the various reference frames and vectors used in the tire model to describe the wheel and tire-road contact. 

The tire model provides a comprehensive kinematic representation of the tire-ground contact point (point B) based on quantities measured or simulated at the actual wheel center (point A_2_). This approach is particularly well suited for the dynamic modeling of suspension systems interacting with the ground.

The kinematic structure relies on a hierarchical decomposition of reference frames, ranging from the ground to the wheel spindle:

The steered cambered frame *R*_2*bis*_, accounting for camber effects:

- The steered non-cambered frame *R_W_*, used to express contact forces and moments without camber;
- The spindle frame *R_2_*, rigidly attached to the suspension system.

The characteristic angles associated with these frames are:

- The camber angle *ε_V_*, between the wheel and the ground normal;
- The self-rotating angle *η_RS_*, representing the wheel’s own rotation about its vertical axis.

The forces and moments generated by the tire model are computed at the contact point B and expressed in the steered non-cambered frame *R_W_*, ensuring consistency with ground contact laws. The ground normal vector, denoted$\;{\vec{N}}_{z}$ is defined in a generic manner to account for irregular or non-planar surfaces.

**Figure 20:**
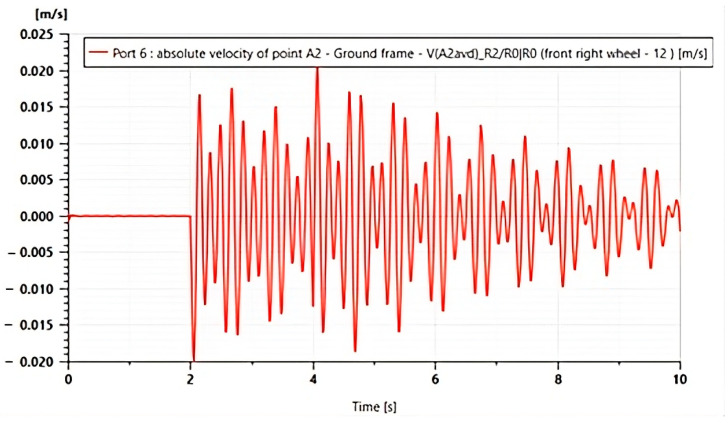
Piezo-Modulated Wheel Vibration: Instantaneous Velocity Decay.

Building upon this framework, Figure 20 presents the time response of the absolute velocity at point A_2_, corresponding to the unsprung mass location on the front right wheel, expressed in a ground-fixed reference frame. This simulation is conducted within the complete vehicle model, which includes a piezoelectric actuator integrated into each suspension at all four wheels.

In the absence of substantial external excitation, the velocity stays near zero before t = 2 s, exhibiting a dynamic equilibrium. An external disturbance, which usually simulates a vehicle crossing an uneven road, causes a transient oscillation in the system starting at around t ≈ 2 s. A damped response is characterized by a gradual decay in amplitude after the maximum velocity of approximately ±0.018 m/s.

**Figure 21:**
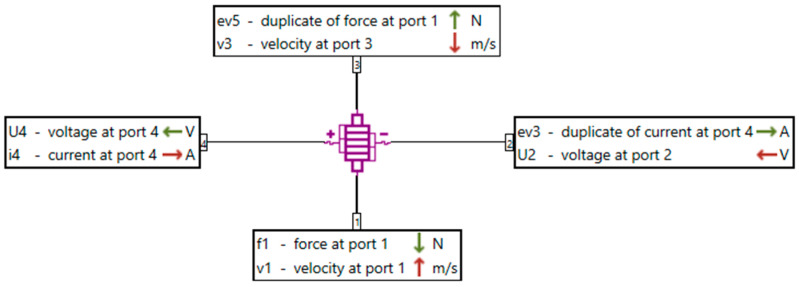
Electromechanical interface of the piezoelectric transducer modeled in Simcenter AMESim.

**Figure 22:**
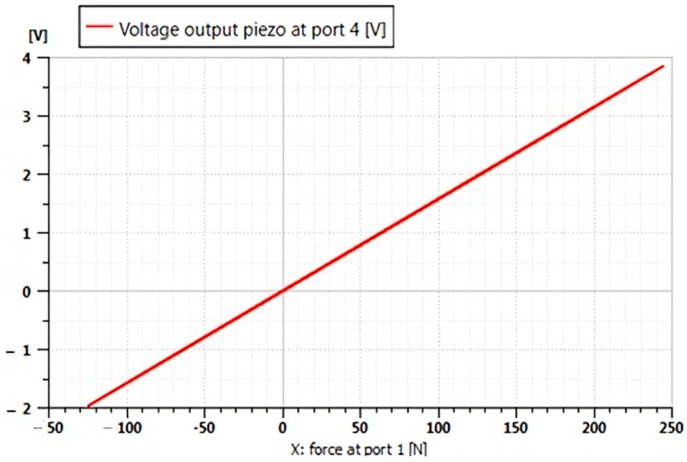
Relationship between applied force (N) and generated voltage (V) of the piezoelectric transducer (output at port 4).

The electromechanical behavior of the piezoelectric transducer is modeled in Simcenter AMESim using the interfaces shown in Figure 21 and Figure 22. Figure 21 highlights the port configuration, where the mechanical quantities (force and velocity) are associated with the upper and lower connections, while the electrical quantities (voltage and current) are represented by the lateral connections. Figure 21 illustrates the multiphysics coupling scheme, emphasizing the role of the duplicated variables (*ev*) in the interaction between the mechanical and electrical domains.

The graph (Figure 22) shows that the Voltage output (Port 4) scales nonlinearly with applied force (Port 1), showing asymmetric sensitivity in tension (+250V) vs. compression (−150 V). Possible suggestions:

- Non-uniform distribution of deformations (for example, bending vs. axial loading).
- Polarization bias in the piezoelectric material (common in PZT ceramics).

**Figure 23:**
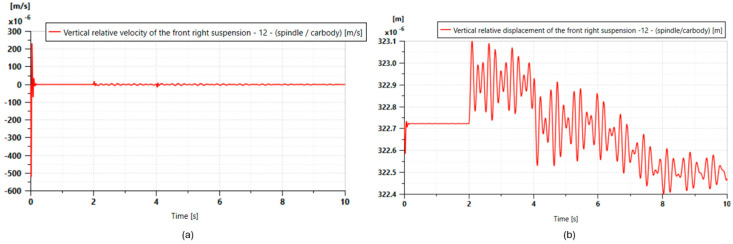
Vertical relative velocity of the front right suspension over time.

Figure 23 illustrates the optimized dynamic response of the suspension: after a significant initial impact (relative speed peaking at −0.6 m/s), the system quickly dampens, stabilizing in less than 5 s and showing minimal residual oscillations. The vertical displacement demonstrates excellent static stability and efficient energy dissipation, with a total variation of 0.7 mm. The precision and speed of stabilization times are two characteristics that ensure comfort and safety while validating the possibility of energy recovery.

To validate our simulation results, we conducted a comparative analysis with experimental findings reported in the literature [24,25]. This benchmarking process demonstrated a high level of consistency in dynamic trends and energy output behavior, reinforcing the reliability and physical relevance of our proposed model.

## 4. Towards Practical Implementation: Signal Conditioning and Energy Storage

Piezoelectric energy harvesting cannot be fully exploited without proper conditioning and storage of the generated power. Indeed, the raw electrical signal from the transducer is inherently unstable, intermittent, and often incompatible with the requirements of low-power embedded systems. Thus, the overall efficiency of a self-powered system depends as much on the mechanical modeling as on the integration of advanced interface circuits capable of maximizing energy extraction and stabilizing the harvested power.

### 4.1. Stability and Conditioning of Electrical Signals

Signal conditioning is a crucial step to convert the alternating electrical oscillations produced by the piezoelectric transducer into a usable form. Conventional methods relying on simple rectification and filtering suffer from significant energy losses and poor adaptability to excitation variations.

Recent work by Al Ghazi et al. (2025) demonstrates that advanced techniques such as Synchronous Electric Charge Extraction (SECE) and Synchronized Switch Harvesting on Capacitors (SSHC) significantly improve energy harvesting efficiency [26]. These methods exploit precise synchronization with mechanical vibrations to optimize energy transfer and reduce internal losses.

In addition, Chen et al. (2023) propose a hybrid dual-mode approach that provides dynamic flexibility to effectively respond to changes in vibration frequency and amplitude, achieving conversion efficiencies exceeding 80% [27]. Moreover, Maximum Power Point Tracking (MPPT) strategies play a key role in dynamically adjusting the circuit impedance to ensure optimal energy extraction in real time.

### 4.2. Storage and Operational Feasibility

Beyond conditioning, energy storage is essential to smooth the power supply to electrical loads and ensure stable autonomy. The use of modern supercapacitors and micro-batteries offers suitable solutions to absorb the intrinsic fluctuations of vibration energy harvesting.

Al Ghazi et al. (2025) and Selleri et al. (2023) highlight recent advancements in integrating these storage devices, particularly ionic-liquid-based supercapacitors, which combine high energy density with durability [26,28]. These components help mitigate power interruptions and provide continuous supply to embedded sensors or modules. This synergy between advanced conditioning and efficient storage constitutes a critical step towards making piezoelectric harvesting systems autonomous, especially in environments with irregular vibrations such as automotive suspensions. Furthermore, these solutions pave the way for industrially viable applications while identifying remaining technological challenges, notably in terms of miniaturization, cost, and long-term reliability.

## 5. Experimental Perspectives

This section presents a comprehensive design of the planned experimental bench, aimed at validating the proposed theoretical models and control strategies under realistic operational conditions.

### 5.1. Instrumentation

The experimental bench will be equipped with high-precision sensors to accurately capture the dynamic response of the system. Key instrumentation includes force sensors designed to measure both input and output forces with a high signal-to-noise ratio, as well as displacement sensors—such as Linear Variable Differential Transformers (LVDT) or laser displacement sensors—capable of recording deflections and relative motions with micron-level accuracy. Additionally, accelerometers will be strategically positioned on critical components to monitor vibrations and transient behaviors. The setup will also incorporate a Data Acquisition System (DAQ) with high sampling rates to ensure the accurate capture of fast transient phenomena without aliasing.

### 5.2. Excitation Profiles According to ISO 8608

The excitation applied to the system will adhere to the guidelines established by ISO 8608, ensuring realistic replication of road surface profiles. This includes generating input signals based on standardized Power Spectral Densities (PSD) corresponding to different road classes. The system will be capable of reproducing both broadband and narrowband excitations to evaluate its response over a wide frequency range. Additionally, precise control of excitation amplitude and frequency content will enable the simulation of diverse operational scenarios.

### 5.3. Measurement Protocols

Measurement protocols will be rigorously defined to ensure both repeatability and reliability of the experimental results. This includes pre-test calibration of all sensors to minimize systematic errors, conducting multiple test runs under identical conditions to evaluate reproducibility, and implementing synchronization mechanisms to temporally align force, displacement, and acceleration data. Additionally, environmental conditions such as temperature and humidity will be continuously monitored to identify and mitigate their potential impact on the measurements.

### 5.4. Data Acquisition and Post-Processing

The data acquisition system will record raw sensor data for subsequent analysis. Sampling rates will be chosen to exceed twice the highest frequency content anticipated in the signals, ensuring compliance with the Nyquist criterion. Real-time monitoring tools will be employed to promptly detect any anomalies during testing. Post-processing will involve noise filtering, spectral analyses such as Fast Fourier Transform (FFT) and Power Spectral Density (PSD) estimation, as well as statistical methods to extract meaningful performance metrics. These experimental strategies provide a solid foundation for a rigorous validation campaign, aimed at bridging the gap between simulation outcomes and real-world applications.

### 5.5. Limitations and Perspectives of Piezoelectric Energy Harvesting

Piezoelectric energy harvesting is currently limited by the internal resistance of materials and circuits, as well as by impedance mismatch, which leads to significant energy losses, particularly under varying vibration conditions. To improve overall efficiency, it is essential to optimize device geometry to enhance electromechanical coupling, select piezoelectric materials with low dielectric losses, and implement adaptive impedance matching techniques such as real-time MPPT. Recent studies, such as those by Ali et al. (2024), have demonstrated the effectiveness of a dynamic system combining a closed-loop converter with a perturbation–observation MPPT algorithm to maximize energy extraction under fluctuating vibration conditions [29]. Similarly, Muscalu et al. (2024) improved energy density by employing MEMS devices based on scandium-doped aluminum nitride, thereby optimizing energy conversion [30]. These combined advances pave the way for reliable and autonomous piezoelectric energy harvesting systems capable of efficient operation in real-world environments with variable excitations.

## 6. Conclusions

The advantages of PZT are that it converts almost 80% of the mechanical energy it receives when it is deformed into electrical energy. In addition, their losses are low, and they have a high Q quality factor (Q greater than 500). Because of their excellent piezoelectric properties (good electromechanical coupling coefficients), PZTs are widely used in sensors and actuators.

In this work, we proposed a vehicle model, where piezoelectric actuators were integrated into the wheel suspension system in parallel. The simulation of the model made it possible to study the response of the piezoelectric as a function of the mechanical energy applied by the vehicle. The conversion of vibrational energy into electrical energy using piezoelectric materials enables embedded energy harvesting applications. The harvested energy can be used to power low-consumption sensors, monitoring units, or active control devices, thereby reducing the energy dependence of certain onboard electronic subsystems. This approach is particularly relevant in the context of connected and autonomous vehicles, where the number of embedded sensors is increasing rapidly [31].

The results corroborate previous work showing the potential of piezoelectric transducers in semi-active or intelligent suspension systems [32,33]. These results will be verified and compared with experimental data in future work. An experimental campaign is already in progress as part of a follow-up project, with the objective of fully validating the proposed model under realistic operating conditions.

## Figures and Tables

**Table 1:** Piezoelectric Material Properties.

Characteristic	Value
Number of wafers	250
Area of wafer	10 × 10 mm^2^
Thickness of a wafer	0.07 mm
$\mathrm{Relative}\;\mathrm{permittivity}\;(K_{33}^{T}$)	2900
Electromechanical coupling factor (k_33_)	0.772
$\mathrm{Elastic}\;\mathrm{constant}\;(s_{33}^{E}$)	2.18 × 10^−11^ m^2^/N

**Table 2:** Mechanical data.

Mechanical Data	Unit	Value
Dimensions (L × W × H)	mm	10 × 10 × 18
Compressive Strength	N/m^2^	8.8 × 10^8^
Tensile Strength	N/m^2^	4.9 × 10^6^
Young’s Modulus	N/m^2^	4.4 × 10^10^
Poisson Ratio	(–)	0.34
Density	kg/m^3^	7900
Weight	grams	16

**Table 3:** Electrical data.

Electrical Data	Unit	Value
Rated voltage (positive only)	VDC	+100
Capacitance	nF	6500
Free deflection	µm	4.9 × 10^6^
Blocked force	N	4.4 × 10^10^
Response time	µs	0.34

**Table 4:** Quarter Car-Model Simulation parameters.

Variable	Unit	Value
Unsprung mass	kg	30
Sprung mass	kg	300
Tire elastic coefficient	N/m	180,000
Passive spring coefficient	N/m	15,000
Tire damping coefficient	Ns/m	100
Passive damping coefficient	Ns/m	1000

**Table 5:** Global model simulation parameters.

Variable	Unit	Value
Car body initial velocity	5	m/s
Wheel mass (front axle)	18	kg
Wheel mass (rear axle)	14	kg
Suspension stiffness (front axle)	21,000	N/m
Suspension stiffness (rear axle)	21,000	N/m
Suspension damping (front axle)	2000	Ns/m
Suspension damping (rear axle)	1500	Ns/m

## Data Availability

The raw data supporting the conclusions of this article will be made available by the authors on request.

## Abbreviations

The following abbreviations are used in this manuscript:

AMESim	Advanced Modelling Environment for performing Simulations
CSS	Car Suspension System
DAQ	Data Acquisition System
DOF	Degree of Freedom
FFT	Fast Fourier Transform
LVDT	Linear Variable Differential Transformer
MEMS	MicroElectroMechanical Systems
MPPT	Maximum Power Point Tracking
PE	Piezoelectric Element
PSD	Power Spectral Density
PZT	Plomb Zirconate Titanate
QCM	Quarter Car Model
SECE	Synchronous Electric Charge Extraction
SSHC	synchronized switch harvesting on capacitors
